# Overexpression of Cx43: Is It an Effective Approach for the Treatment of Cardiovascular Diseases?

**DOI:** 10.3390/biom15030370

**Published:** 2025-03-04

**Authors:** Kerstin Boengler, Beatrice Mantuano, Shira Toledano, Ofer Binah, Rainer Schulz

**Affiliations:** 1 Institute of Physiology, Justus-Liebig University, 35392 Giessen, Germany; 2 Department of Clinical and Biological Sciences, University of Torino, 10125 Torino, Italy; 3 Department of Physiology, Biophysics and Systems Biology, Rappaport Faculty of Medicine, Technion-Israel Institute of Technology, Haifa 3190601, Israel

**Keywords:** connexin, gap junction, mitochondria, hemichannel, cardiac disease

## Abstract

In the heart, Connexin 43 (Cx43) is involved in intercellular communication through gap junctions and exosomes. In addition, Cx43-formed hemichannels at the plasma membrane are important for ion homeostasis and cellular volume regulation. Through its localization within nuclei and mitochondria, Cx43 influences the function of the respective organelles. Several cardiovascular diseases such as heart failure, ischemia/reperfusion injury, hypertrophic cardiomyopathy and arrhythmias are characterized by Cx43 downregulation and a dysregulated Cx43 function. Accordingly, a putative therapeutic approach of these diseases would include the induction of Cx43 expression in the damaged heart, albeit such induction may have both beneficial and detrimental effects. In this review we discuss the consequences of increasing cardiac Cx43 expression, and discuss this manipulation as a strategy for the treatment of cardiovascular diseases.

## 1. Localization and Function of Cx43 Within the Healthy Heart

The connexin protein family consists of 20 or 21 members in mice and humans [1,2], and connexins are named according to their approximate molecular weight of 20–62 kDa. In humans, connexin expression is mapped to more than 110 cell types, such as heart, brain and liver [3]. In addition to their wide distribution, connexin expression is highly dynamic, with temporal changes within cells and tissues, and specific combinations of expressed connexin isoforms due to the regulation of transcription and epigenetic mechanisms [4,5]. In the heart, Cx40 is mainly found in atrial cardiomyocytes, and, like Cx45, it is present in the atrioventricular node, the His-bundle and the ventricular conduction system. Cx45 is also localized in the sinoatrial node [6], and Cx30.2 in the conduction system [7]. In the present article, we focus on the expression and function of Cx43, which is encoded by the *Gja1* gene and ubiquitously expressed in the heart, as well as in over 50 organs and many cell types, such as astrocytes, retinal cells, endothelial cells and alveolar cells [3,8,9,10,11].

Connexins are typically composed of intracellular N-terminal and C-terminal regions, four transmembrane domains, and two extracellular and one intracellular loops, and are synthesized in the endoplasmic reticulum; six connexins oligomerize into a connexon or hemichannel in the Golgi or trans-Golgi network, and traffic in vesicles along the microtubules to the plasma membrane [12,13]. Docking of two hemichannels from neighboring cells results in the formation of gap junctions, which mediate intercellular electrical and chemical cell–cell coupling [14].

Six identical connexins oligomerize into a homomeric hemichannel or connexon. Connexons consisting of two or more connexin isoforms are called heteromeric. Docking of hemichannels from adjacent cells results in the formation of homotypic (composed of two identical connexons), heterotypic (most typically composed of two different homomeric connexons), or heteromeric (composed of two different heteromeric connexons) connexin channels [9,15]. Accordingly, the different connexin channels display specific unitary conductance, charge selectivity and pore diameters [16,17]. In addition to the originally described function of connexins as gap junction proteins [14,18], recent studies suggest that connexins have additional functions. For example, Cx43 is detected within extracellular vesicles of different cellular origin [19,20,21,22], such as H9C2 cells [20], and phosphorylated Cx43 controls exosome release [23]. Thus, Cx43 not only contributes to the communication of neighboring cells, but also to that of cells located at greater distances. Non-paired hemichannels exist in the plasma membrane and, when open, allow communication with the extracellular space, including transporting molecules with a molecular weight of up to 1.2 kDa [24]. Whereas such hemichannels are predominantly closed under physiological conditions, they open under certain pathophysiological situations, such as in *mdx* mice (having a point mutation in the *Dmd* gene; a common mouse model of Duchenne muscular dystrophy), right ventricular arrhythmogenic cardiomyopathy in Plakophilin-2-(*Pkp2*)-deficient mice, and in ischemia/reperfusion (I/R) injury in mice [25,26,27]. Another subcellular location where Cx43 is detected are the mitochondria. However, cardiac Cx43 is not localized within all mitochondria, but is mainly present in subsarcolemmal mitochondria and not in interfibrillar mitochondria [28,29,30,31]. The subsarcolemmal mitochondria are located beneath the sarcolemma and differ from interfibrillar mitochondria in form and function [32,33]. The analysis of subsarcolemmal mitochondria shows that Cx43 is present in the inner mitochondrial membrane [34,35]. Regarding the orientation of Cx43 within the inner membrane, it is suggested that the C-terminus is facing the intermembrane space [28,36]. Within mitochondria, Cx43 regulates several functions, including respiration [37], potassium uptake [38], formation of reactive oxygen species [39] and opening of the mitochondrial permeability transition pore [35,40]. Recent data show that in addition to mitochondria, Cx43 is also found in nuclei of rat and mouse cardiomyocytes [41], as well as in nuclei of colon samples from patients with ulcerative colitis [42]. Whereas the pathways by which Cx43 reaches the nuclei are presently unclear, diffusion is excluded. Finally, Cx43 forms channels in the nuclear envelope [41] and influences gene expression in astrocytes [43] and cardiomyocytes [41].

The complexity of Cx43-mediated functions is not only evident in that it has several subcellular localizations, but also by the fact that in addition to the 43 kDa full-length protein, alternative translation generates a truncated 20 kDa form of Cx43 [44]. This N-terminally truncated form, termed Gja21-20k, is detected in different cell types and organs including the heart [44,45,46]. Gja1-20k is found within the Golgi apparatus and mitochondria. Like the full-length Cx43, mitochondrial Gja1-20k localizes to subsarcolemmal mitochondria [31], but unlike the 43kDa form, it is not found within the inner, but within the outer mitochondrial membrane [47]. Mitochondrial Gja1-20k affects their function in several aspects, including respiration, membrane potential and biogenesis [45,47]. Additionally, Gja1-20k influences oligomerization of hexamers, stabilization of actin filaments and the transport of Cx43-formed hexamers to the plasma membrane [48,49]. Moreover, Gja1-20k expression correlates with the intercellular transport of mitochondria from mesenchymal stromal cells to chondrocytes undergoing oxidative stress, a process suggested to contribute to tissue healing in degenerative diseases such as osteoarthritis [50]. The decisive role of Gja1-20k in the maintenance of cardiac function is demonstrated in Gja1-20k-deficient mice, which have reduced protein levels of full-length Cx43, abnormal cardiac conduction and a significantly reduced lifespan [51].

Cx43 function is regulated by post-translational modifications such as glycosylation, N-acetylation, S-nitrosylation, ubiquitination and phosphorylation (for review, see [52]). It is suggested that the cysteine residues in the extracellular loops function as redox sensors, thereby controlling connexin function [53]. Respecting Cx43 phosphorylation, the 21 amino acids (mainly serine and tyrosine residues) targeted by different protein kinases are located in the carboxy-terminus of the protein. Kinases phosphorylating Cx43 include protein kinase B (AKT, targets serine 373), protein kinase C (PKC, targets serine 368), casein kinase 1 (CK1, targets serines 325, 328, 330), mitogen-activated protein kinase (MAPK, targets serines 262, 279, 282) and pp60src kinase (targets tyrosine 247 and 265) (for review, see [54]). Cx43 phosphorylation is important for the regulation of its transport and assembly into gap junctions, gating of Cx43-formed channels and gap junction turnover [3,54]. In addition to the function of gap-junctional Cx43, the activities of hemichannels and mitochondria are also regulated by phosphorylation [25,55,56]. Whether Cx43 phosphorylation is a prerequisite for its import into mitochondria, or whether the protein is phosphorylated within the organelle by mitochondrially localized kinases, is presently unclear [57]. Nuclear Cx43 is also phosphorylated, thereby affecting its activity in the outer nuclear membrane [41]. Whereas the aforementioned studies show that phosphorylation controls Cx43 activity in the subcellular compartments, it is completely unknown whether there is an interplay between the phosphorylation sites in the specific compartments. The analysis of mitochondria isolated from mice with mutated Cx43 phosphorylation sites demonstrates alterations in the phosphorylation status of additional amino acids [55], showing that lack of phosphorylation at one amino acid influences the phosphorylation at other residues. The consequences of such secondary changes in Cx43 phosphorylation are yet to be investigated. Furthermore, it is unclear whether the interactions between the individual Cx43 phosphorylation sites are restricted to the mitochondria or whether they also occur in other compartments where Cx43 is localized. It needs to be investigated if Cx43 is phosphorylated in a specific pattern in the individual compartments, and if a dynamic interplay in the phosphorylation at the specific amino acids occurs in physiological as well as in pathological conditions.

Another approach to control Cx43 function is to adjust the amount of the protein, determined by the fine balance between synthesis and degradation. The half-life of cardiac Cx43 is relatively short (1–2 h) [58,59], implying that gap junctions are renewed several times/day [9] due to transcriptional modulation [60]. In addition, the epigenetic machinery regulates connexin expression via DNA methylation, histone acetylation or micro-RNAs [61,62]. Removal of gap junctions from the plasma membrane involves the internalization of gap junction plaques and the subsequent formation of annular gap junctions, also termed connexosomes [63]. Annular gap junctions are degraded via autophagosomal and endolysosomal pathways, whereas a fraction of newly synthesized Cx43 undergoes proteasomal degradation via ERAD (endoplasmic reticulum-associated degradation) [64,65]. Cx43 degradation is classically triggered by protein ubiquitination [66].

Taken together, knowledge about Cx43 function extends beyond its role in intercellular communication via gap junctions and hemichannels. Cx43 contributes to cell–cell communication also via its presence within exosomes. Furthermore, Cx43 is located within nuclei and mitochondria, and influences the function of the respective organelles. Whereas Cx43 participates in the regulation of cell functions via its specific localization, its own function is also controlled via alternative translation and post-translational modifications. As a protein with a short half-life, an additional aspect to control Cx43 function is transcriptional regulation, protein turnover and degradation. All these aspects highlight the wide range of options used to regulate Cx43 function under physiological and pathological conditions.

## 2. Cx43: Expression, Localization and Function in the Diseased Myocardium

Considering the essential role of Cx43 in the regulation of cardiac function, it is necessary to understand the changes in Cx43 activity associated with cardiovascular diseases such as hypertension, hypertrophy, myocardial infarction, heart failure and arrhythmias. The present review summarizes only general aspects of Cx43 dysfunction in cardiovascular diseases; for a more detailed description of these changes occurring in specific pathological conditions, please see previously published articles [6,67,68,69,70,71,72,73]. Despite the different pathomechanisms underlying the abovementioned diseases, there are several aspects of Cx43 dysregulation shared by these diseases. In hypertrophic heart disease, early stages are often associated with increased Cx43 expression, whereas decreased expression is reported in later (decompensated) stages [6,73,74]. Myocardial remodeling caused by hypertension also shows such two-step process in Cx43 expression; an upregulation in the compensated stage and a downregulation in the decompensated stage [74]. Reduced Cx43 expression is also evident in hearts with dilated and ischemic cardiomyopathy, following myocardial infarction and in failing hearts [6,72,75,76,77]. Cx43 degradation during I/R in rat hearts in vitro correlates with the induction of autophagy [66]. In addition, micro-RNA-1 plays a role in the regulation of Cx43 function and localization in cardiac hypertrophy in mice [61], in the protection from ischemic damage by telmisartan in rat myocardium [62] and in mice iPSC-CMs (induced pluripotent stem cell-derived cardiomyocytes) subjected to rapid pacing [78]. Cardiovascular diseases influence alternative Cx43 translation; for instance, in a mouse model of arrhythmogenic cardiomyopathy, the amount of Gja1-20k is reduced [79]. Furthermore, spontaneously hypertensive rats display diminished levels of Gja1-20k [45], whereas in mouse hearts undergoing I/R, the amounts of Gja1-20k are enhanced [47]. The quantity of Gja1-20k, in turn, may control the quantity of full-length Cx43; for example, in mice with mutated internal translation initiation sites, the levels of Cx43 are decreased [51]. The reduction in Cx43 can result in diminished intercellular coupling, and thereby to a decrease in conduction velocity. The influence of Cx43 on the conduction velocity is dependent on the extent of Cx43 protein reduction; a 50% reduction decreased conduction velocity by 44% in one study [80], but was without an effect in another study [81]. A further reduction to only 10% of baseline Cx43 leads to a ~33% decrease in the transverse conduction velocity [82]. Studies in which a specific contribution of mitochondrial Cx43 towards cardiac pathologies are investigated are scarce [56]; thus far, only a decrease in mitochondrial Cx43 in a rat model of dilated cardiomyopathy is described [83].

In general, Cx43 regulates a variety of cellular functions, such as conduction velocity, mitochondrial function [56], alterations in transcriptomics [43,84] and selective incorporation of micro-RNAs into extracellular vesicles [85]; all of these functions can be impaired by a reduction in Cx43 occurring in pathological conditions. In addition to the adverse consequences of reduced Cx43 in cardiac pathologies, at intercalated discs and probably also at other subcellular localizations, Cx43 lateralization at the plasma membrane alters Cx43 function and that of the cardiomyocytes. In the physiological situation, following Cx43 synthesis in the endoplasmic reticulum and oligomerization in the trans-Golgi network [86], the protein is directed to the intercalated discs in a process involving microtubules and actin filaments [87,88]. In pathological situations, there is often a shift of Cx43 from the intercalated discs to the lateral sides of the cardiomyocytes. Such lateralization occurs in several pathological states such as hypertension [89,90,91,92], post-myocardial infarction [93], and in hypertrophic, dilated and ischemic cardiomyopathy [6]. Lateralized Cx43 lowers the conduction velocity, thereby contributing to the development of arrhythmias [6], albeit there seems to be a large conduction reserve [94]. Hemichannels formed by lateralized Cx43 are activated under cardiac stress, which promotes the occurrence of arrhythmias and sudden death [95]. Data on the shift of Cx43 in cardiovascular pathologies at subcellular localizations other than the plasma membrane, such as mitochondria, nuclei and exosomes, are scarce. In addition to its amount and localization, Cx43 function is controlled by post-translational modifications such as phosphorylation. Interestingly, Cx43 phosphorylation also regulates the amount of Cx43 protein. In mice in which amino acids targeted by PKC, MAPK or CK1 are mutated to non-phosphorylatable residues, Cx43 expression is reduced [55]. However, mutation of the residues targeted by CK1 to phosphatase-resistant glutamic acids in human iPSC-CMs leads to decreased Cx43 expressions despite a tendency towards increased Cx43 mRNA levels [96]. The differences between the data relating to the consequences of mutated CK1-target sites may be explained by the different maturation stages of the cardiomyocytes.

Cx43 phosphorylation triggers its modification with lysine 63 (K63)-linked polyubiquitin chains and subsequent Cx43-internalization [97], with PKC being involved in Cx43 ubiquitination [98]. AKT is also implicated in this process, since the mutation of serine 373 to alanine renders Cx43 resistant to ubiquitination [66]. The kinases Src and ERK (extracellular-signal regulated kinase) and Cx43-S262 phosphorylation regulate connexosome formation [99,100]. In addition to the influence of Cx43 phosphorylation on its degradation, Cx43 phosphorylation status of Cx43 at specific residues is important for its lateralization. The mutation of residues targeted by CK1 to phospho-mimicking glutamic acids prevents Cx43 lateralization in a mouse model of Duchenne muscular dystrophy [25]. In the physiological situation, mutation of S368 to alanine does not induce a Cx43 shift away from the intercalated discs [101]. However, increased Cx43 lateralization in cardiomyocytes is associated with a dephosphorylation of the protein in cardiovascular diseases such as ischemia [102], heart failure [103,104] and hypertrophy [105]. In different cardiac diseases, Cx43 phosphorylation is altered such that the phosphorylation at serine 368 by PKC varies [106]. Cx43 phosphorylation at serine 368 by PKC influences Cx43 stability via conformational changes of Cx43-formed channels, which affect the channel permeability. Moreover, serine 368 phosphorylation influences Cx43 internalization and its degradation, leading to reduced intercellular communication [106]. Serine 373 targeted by AKT becomes increasingly phosphorylated in MDCK43 cells following 5–30 min of hypoxia, which contributes to the elimination of the protein–protein interaction between Cx43 and zonula occludens 1, in turn leading to increased gap junction size and intercellular communication. Following 60 min of hypoxia, serine 373 phosphorylation returns to control levels [107]. Increased gap junction size induced by serine 373 phosphorylation may indicate the first step in gap junction disassembly [54]. Phosphorylation at serines 325/328/330, which are targeted by CK1, is reduced in Chagas disease cardiomyopathy [108] and following ischemia in mouse hearts [109]. The amount of lateralized Cx43 increases following 30 min of ischemia in isolated mouse hearts, whereas lateralized Cx43 is not phosphorylated at serines 325/328/330, and the consequent reduced functionality of gap junctions may prevent aberrant cell–cell communication [109]. The loss of cardioprotection by ischemic preconditioning in mice with mutated serines 325/328/330 demonstrates the importance of these amino acids for myocardial I/R injury [55]. However, the influence of increased phosphorylation at serine 262, as well as decreased phosphorylation at serine 365 and 368, described in the mouse strain with mutated serines at positions 325/328/330 in the context of ischemic preconditioning, is presently unclear [55]. Ischemic preconditioning prevents the ischemia-induced dephosphorylation of Cx43 [110]. Regarding the importance of serine 282, it is shown that the mutation of this residue to alanine causes spontaneous arrhythmias in mice and rats [111,112]. Furthermore, mice with serines 325/328/330 mutated to alanine are susceptible to arrhythmias, whereas the mutation of these serines to phosphomimetic glutamic acids results in a diminished susceptibility to ventricular arrhythmias [113]. Generally, Cx43 dephosphorylation is associated with the occurrence of arrhythmias in heart failure and myocardial ischemia [114]. Details on the alterations of Cx43 phosphorylation in cardiac diseases such as I/R injury, heart failure, arrhythmia, hypertension and hypertrophy are reviewed elsewhere [115]. Importantly, the phosphorylation status of Cx43 is not only controlled by kinases, but also by phosphatase activity [116], and a dysregulation of protein phosphatases occurs in heart failure, cardiac ischemia and atrial fibrillation [114].

**Figure 1:**
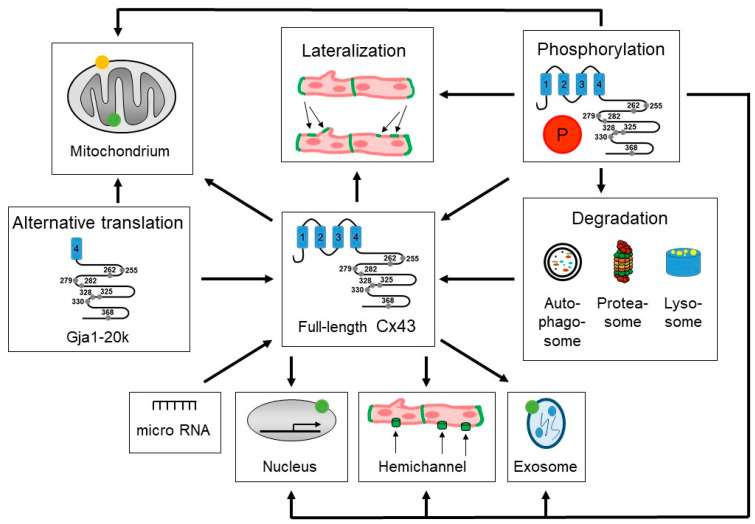
Interplay between factors regulating amount and localization of Cx43. The Figure illustrates the relationships between factors important for the localization and the amount of Cx43 within cardiomyocytes, and therefore for Cx43-mediated function. The numbered blue boxes (1–4) in the schematic representation of Cx43 indicate transmembrane domains. The green dots represent Cx43 within mitochondria (inner mitochondrial membrane), nuclei and exo-somes, and the orange dot represents Gja1-20k within mitochondria (outer mitochondrial mem-brane). The numbered gray dots represent the positions of some amino acids, which can be phosphorylated by protein kinases. The arrows indicate the interactions between Cx43 localizations and processes that regulate Cx43 function. Please note that the arrows only indicate which factor has an influence on another factor and not whether this influence is activating or inhibiting. For details of how the individual parameters relate to each other in terms of Cx43 localization and function, see the text and tables.

Collectively, cardiovascular pathologies are associated with Cx43 dysregulation, including decreased protein amounts, enhanced Cx43 lateralization at the plasma membrane, a modification of its phosphorylation status and changes of alternative translation. These parameters, which are crucial for the regulation of Cx43 function, show a variety of interactions that indicate a complex control of Cx43 function. The interactions of factors that contribute to the regulation of Cx43 amount and localization are summarized in Figure 1.

The Figure illustrates the relationships between factors important for the localization and the amount of Cx43 within cardiomyocytes, and therefore for Cx43-mediated function. The numbered blue boxes (1–4) in the schematic representation of Cx43 indicate transmembrane domains. The green dots represent Cx43 within mitochondria (inner mitochondrial membrane), nuclei and exosomes, and the orange dot represents Gja1-20k within mitochondria (outer mitochondrial membrane). The numbered gray dots represent the positions of some amino acids, which can be phosphorylated by protein kinases. The arrows indicate the interactions between Cx43 localizations and processes that regulate Cx43 function. Please note that the arrows only indicate which factor has an influence on another factor and not whether this influence is activating or inhibiting. For details of how the individual parameters relate to each other in terms of Cx43 localization and function, see the text and tables.

## 3. Overexpression of Cx43 as a Therapeutic Approach for the Treatment of Cardiovascular Diseases

The fundamental contribution of Cx43 to cardiac function makes it a potential target for the treatment of cardiovascular diseases. Pharmacological modulation of gap junctions and/or hemichannels can be accomplished by targeting various aspects of Cx43 function, including trafficking, channel gating and assembly or disassembly of gap junctions [12]. Whereas several chemicals (e.g., heptanol, carbenoxolone and 18α-glycyrrhetinic acid) affect Cx43-formed gap junctions or hemichannels [12], less information is available on their effects on Cx43 at other subcellular localizations such as mitochondria or nuclei. Peptide molecules (e.g., Gap19, Gap26, Gap27 or AAP10) are generally considered more specific than these chemicals, and are shown to interact with Cx43 or mimic its function, and can inhibit hemichannel function alone (Gap19) or also gap junctions (Gap26, Gap27) [117]. Analyzing the effects of these peptides which inhibit hemichannels while preserving gap-junctional communication reveals that they constitute a promising approach for treating heart diseases [118]. For detailed reviews summarizing the use of such peptides in cardiovascular pathologies, the reader is referred to references [119,120].

The demonstration of reduced Cx43 protein in several cardiovascular diseases (see Chapter 2) implies that overexpressing cardiac Cx43 may induce beneficial effects in the protection against these pathological conditions. To investigate the consequences of enhanced Cx43 expression in the physiological situation, transgenic mice were generated in which Cx43 is constitutively expressed under control of the cytomegalovirus promoter [121]. These mice show enhanced junctional communication in dye-coupling experiments, and although generally viable and fertile, have a reduced postnatal viability. Detailed analysis indicates that hearts of the Cx43-overexpressing mice have morphological defects in the right ventricle and the pulmonary outflow tract, as well as abnormalities in the coronary vasculature. The reduced viability and the malformations detected in these mice demonstrate that overexpressing Cx43 can trigger harmful effects. The mouse line described above is the only transgenic line published to date in which constitutive overexpression of Cx43 was achieved. Further studies, aimed at investigating the role of Cx43-overexpression, without using genetically modified animals, are required.

In this regard, Cx43 overexpression in iPSC-CMs results in enhanced Cx43 amounts, specifically at the sarcolemma, and augmentation of voltage-gated Na^+^-channel expression, as well as channel recruitment to the sarcolemma [122]. In turn, action potential upstroke velocity increases, which consequently leads to enhanced intercellular coupling. In a porcine model of atrial fibrillation induced by rapid pacing, adenoviral Cx43 gene transfer increases its protein levels and prevents the development of persistent atrial fibrillation [123]. Furthermore, it is shown that adenoviral Cx43 overexpression does not affect atrial conduction (P-wave duration and intra-atrial conduction time) in control animals, whereas it prevents atrial fibrillation in porcine myocardium [124]. In addition to its anti-arrhythmic effects, adenoviral Cx43 gene transfer enhances atrial Cx43 levels and prevents its lateralization, as evident in atrial fibrillation [124]. Injecting Cx43-expressing adenoviruses to the healed scar border of infarcted porcine myocardium improves conduction velocity and decreases ventricular tachycardia [125]. Whereas the amount of Cx43 increases following adenoviral gene transfer, the percentages of phosphorylated Cx43 and Cx43 located at the intercalated discs are similar between endogenous Cx43 and adenovirus-derived Cx43. In addition, injecting Cx43-expressing lentivirus in cryo-injured myocardium increases Cx43 expression and diminishes the occurrence of ventricular tachycardia in the post-infarct scar; this effect is most likely due to enhanced heterotypic cell–cell electrical coupling between the native myocardium and the infarct area [126].

The engraftment of Cx43-expressing skeletal myoblasts in mouse myocardial infarcts induced by cryoinjury protects against ventricular tachycardia [127]. However, these positive effects are not confirmed in a similarly structured study in infarcted rat hearts, in which the myoblasts are not from transgenic mice expressing Cx43 under the control of a skeletal muscle promoter, but are derived from cells transduced with a Cx43-carrying lentivirus [128]. The differences between these two studies may be explained by the different animal models and the level of Cx43 overexpression in the transplanted cells. In another attempt to treat myocardial infarction by cell transplantation, the delivery of mesenchymal stem cells combined with native or Cx43-overexpressing human skeletal myoblasts into the post-infarction mouse heart indicates no effect of Cx43 overexpression on the left or right ventricular ejection fraction or left/right ventricular mass [129]. Since (as mentioned above) there is an increase in both the overall amount of Cx43 and its lateralization in *mdx* mice (a common mouse model of Duchenne muscular dystrophy) and in human Duchenne hearts [95], it is proposed that Cx43 normalization (i.e., Cx43 downregulation), will alleviate cardiac dysfunction in this pathology. Indeed, crossing *mdx* mice with heterozygous Cx43-deficient mice decreases Cx43 expression to control values, reduces Cx43-lateralization and prevents the development of arrhythmias induced by an isoproterenol administration [130].

In porcine hearts in vivo, Cx43 translocation into mitochondria is associated with reduction of infarct size by ischemic preconditioning [131]. The cytoprotection by mitochondrial Cx43 is confirmed in stem cell antigen-1^+^ cells transfected with Cx43 fused to a mitochondrial targeting sequence [132]. Such a transfection increases the amounts of Cx43 at the inner mitochondrial membrane, reduces apoptosis and improves cell survival following oxygen–glucose deprivation. Augmenting the amount of Cx43 in HL1 cells by transfection leads to enhanced respiration [37]. However, the overexpression of mitochondrial Cx43 by injecting adeno-associated virus serotype 2/9 in mice induces mild diastolic dysfunction and increases the susceptibility to ventricular arrhythmia indicated by QTc, QT interval and JT interval prolongation [133]. Furthermore, doxorubicin-induced cardiotoxicity in H9C2 cells, which enhances the formation of reactive oxygen species (ROS), is characterized by increased amounts of mitochondrial Cx43 [134]. Therefore, these findings demonstrate that enhanced levels of mitochondrial Cx43 are not involved per se in protective signaling pathways; rather, the effects mediated by mitochondrial Cx43 seem to be dependent on the actual context (e.g., species, cell type, physiological or pathological situation) in which they are studied. A contribution of mitochondrial Gja1-20k to the cardioprotection by ischemic preconditioning is suggested, since Gja1-20k accumulates in mitochondria following a preconditioning stimulus [47]. The treatment of mice with adenoviruses to stimulate the Gja1-20k expression reduces ROS formation and infarct size following I/R induced by occlusion of the left anterior descending artery (LAD) in vivo as well as in Langendorff-perfused isolated mouse hearts [47]. Therefore, enhanced amounts of Gja1-20k in mouse hearts are associated with a cardioprotective phenotype. Table 1 summarizes studies in which direct effects of Cx43- or Gja1-20k-overexpression in cardiovascular cells are characterized.

There are several approaches for treating cardiovascular diseases which do not directly target Cx43, but are associated with changes in Cx43 amounts, and may therefore alter myocardial function. The results of such studies related to Cx43 expression and localization are summarized herein. Isolated rat hearts undergoing hypothermic I/R show an upregulation of miR-17, present increased apoptosis and develop arrhythmias. These alterations are associated with reduced amounts of Cx43. In contrast, miR-17 inhibition increases the conduction velocity and reduces arrhythmias. Inhibition of miR-17 in rat hearts undergoing hypothermic I/R is accompanied by enhanced Cx43 expression, al-though the amounts of Cx43 protein are significantly lower than in control hearts [135]. In H9C2 cells, hypoxia/reoxygenation (H/R) downregulates Cx43; the addition of lactate prior to H/R normalizes the Cx43 level to that of normoxic control cells [136]. In normoxic H9C2 cells, lactate augments the amounts of Cx43 mRNA and protein—an effect mediated by binding of the transcription factor EB to the Cx43 promoter. Ischemic cardiomyopathy in rats caused by 4-week LAD ligation decreases Cx43 expression, an effect counteracted by overexpressing caveolin-1, presumably via decreased phosphorylation of the tyrosine kinase cSrc [137]. Furthermore, I/R of isolated rat hearts reduces the Cx43 amount, which is partially prevented by injecting C2C12 cell-derived exosomes into the rat jugular vein prior to I/R [138]. A single targeted heavy ion radiation to rabbit hearts increases Cx43 at the intercalated discs and at the lateral sides of the cardiomyocytes, lasting for at least one year [139]. Despite the conceivable negative effects of Cx43 lateralization, no changes are detected in the ECG or echocardiogram up to one year post-radiation. These data indicate that in this model, Cx43 lateralization does not adversely affect cardiac function. In fact, with heavy ion radiation two weeks post-myocardial infarction (MI) in rabbit hearts in vivo, the moderate increase (~30% above control) in Cx43 and its lateralization are associated with reduced cardiac vulnerability to arrhythmias [140].

Rats treated with monocrotaline to induce pulmonary hypertension demonstrate Cx43 downregulation in right atrial tissue. The administration of the TRPV2 (transient receptor potential vanilloid type 2) inhibitor tranilast prevents the monocrotaline-stimulated remodeling of the right atrium and enhances Cx43 expression, while the amount of Cx43 is stabilized below the control level [141]. Another study describes the protective effects of monoterpene 1,8-cineole in monocrotaline-induced pulmonary hypertension, which are related predominantly to the prevention of Cx43-lateralization, rather than to the inhibition of Cx43 downregulation [92]. In contrast, pulmonary hypertension induced by chronic hypoxia promotes Cx43 expression in rat pulmonary arteries [142]. Moreover, enhanced amounts of Cx43 are detected in primary and third-level branches of mesenteric arterioles of spontaneously hypertensive rats [143].

Heart failure induced by transverse aortic constriction (TAC) in mice is accompanied by decreased Cx43 expression. The administration of carnosol (a phenolic compound found in rosemary) enhances Cx43 expression without attaining the levels in control animals, and reduces the vulnerability towards ventricular fibrillation [144]. Whereas Cx43 decreases in heart failure caused by high pacing in dogs, renal denervation increases its amount, yet below the control level [77]. In a heart failure model with preserved ejection fraction in mice induced by high-fat diet and L-NAME administration, Cx43 levels decline. This effect is partially reversed by the calcium-sensitizing drug levosimendan [145]. Cardiac remodeling induced by isoproterenol is associated with Cx43 downregulation, which is normalized to control levels by the administration of pinocembrin, a flavonoid with antioxidant and anti-inflammatory effects [146]. Pinocembrin also reduces the susceptibility to ventricular fibrillation.

Collectively, reduced Cx43 expression is a common feature of several cardiovascular diseases, although some pathological conditions such as the early stages of hypertrophy and hypertension are accompanied by enhanced Cx43 amounts. Accordingly, it is proposed that treating heart diseases associated with decreased Cx43 can be accomplished by enhancing Cx43 expression. Hence, whereas in some cases, Cx43 levels stagnate below those of respective controls, other settings demonstrate a complete restoration of Cx43 amounts. Of note, only one study describes Cx43 amounts above the control level, whereas all other studies report, at best, its normalization. Table 2 summarizes the studies in which therapeutic approaches to treat cardiovascular diseases do not directly target Cx43, yet are nevertheless associated with changes in Cx43 levels and/or Cx43 function. The specific interventions and their effects on Cx43 are provided in Table 2.

## 4. Conclusions

A common feature of several cardiovascular diseases is that they are associated with decreased amounts of Cx43. However, as biphasic responses (a short-term increase followed by a long-term decrease) evolve towards the pathological state, the conclusion as to whether up- or downregulation of Cx43 occurs is dependent on the time point analyzed. Further, some pathological conditions are associated with increased Cx43, implying that in these cases, reducing Cx43 levels can constitute a beneficial therapeutic approach. Importantly, in cardiovascular diseases associated with decreased Cx43, it should be noted that in most cases, the downregulation is shown only for total Cx43 (as mostly shown by Western blot analysis) or for gap-junctional Cx43 (mostly shown by immunohistochemistry). Such analysis does not take into account other localizations at which Cx43 is present, such as hemichannels, mitochondria, nuclei and exosomes, for which a specific Cx43 reduction in a certain pathological condition is possible, and thus should be determined. When considering the treatment of cardiovascular diseases associated with decreased Cx43, increasing Cx43 might be a beneficial therapeutic strategy. It is important to ensure that the Cx43 level is enhanced only in the cells and/or compartments where it was originally reduced. This approach takes into account that increasing Cx43 expression in sites presenting normal levels may induce adverse effects such as Cx43 lateralization, which may constitute a pro-arrhythmic substrate. Additionally, increased mitochondrial and nuclear Cx43 may adversely affect cellular energetics and gene expression, respectively. The complexity regarding the increase in mitochondrial Cx43 is further augmented by the fact that in cardiomyocytes, Cx43 is only localized in subsarcolemmal and not in interfibrillar mitochondria [56], and therefore Cx43 overexpression should specifically affect this mitochondrial subpopulation. It is noteworthy that only one of the abovementioned strategies for treating cardiovascular diseases is associated with increased Cx43 protein that exceeds the respective control group. Thus, it is unclear whether reaching the control values represents the maximal possible increase, or whether the amount of the protein is specifically stabilized at this level. Possibly, attaining the normal Cx43 levels and not beyond, in situations with decreased amounts of Cx43, is more beneficial than overexpressing Cx43, thereby preventing the adverse effects of excessive Cx43. It remains to be established whether modulation of Cx43 expression via interfering with micro-RNAs or protein degradation prevents Cx43 overexpression, thereby contributing to the Cx43 stabilization at control values. Accordingly, it will be advantageous to investigate the contribution of micro-RNAs and protein degradation to Cx43 regulation, and thus discover additional targets for controlling Cx43 expression. Additionally, since alternative translation of Cx43 is important for regulating the amount of full-length Cx43, its contribution towards normalization of Cx43 amounts in the treatment of cardiovascular diseases needs to be further investigated. Therefore, the analysis of factors affecting Cx43 levels under conditions in which Cx43 is gradually increased is highly relevant. The use of Cx43 as a therapeutic target in cardiovascular diseases associated with reduced Cx43 levels should not only ensure the increase in Cx43 protein levels; rather, Cx43 function should also be controlled in terms of its trafficking to and assembly into gap junctions, gating of Cx43-formed channels, functional control of mitochondria, nuclei and exosomes, as well as Cx43 degradation. Finally, when considering increasing Cx43 expression in the diseased myocardium, a physiological phosphorylation of the protein needs to be ensured to prevent aberrant Cx43 function.

In summary, the presence of reduced Cx43 levels in diseased myocardium suggests that Cx43 overexpression can represent a therapeutic approach for the treatment of such diseases; indeed, beneficial effects elicited by increased Cx43 protein amounts are demonstrated. However, it should be taken into consideration that Cx43 overexpression may be detrimental in the developing heart (as shown by constitutive Cx43 overexpression in transgenic mice) and that some cardiovascular diseases are associated with enhanced Cx43 levels. Thus, a moderate increase in Cx43 to physiological levels may be more effective in the treatment of cardiovascular disease than excessive overexpression, as this may circumvent potential negative effects of increased Cx43 amounts. The complex regulation of Cx43 through its different localizations in cellular compartments, phosphorylation, alternative translation or degradation necessitates tight control of its function at multiple levels. Accordingly, a detailed analysis of the parameters modulated by Cx43 is required to fully assess the consequences of increased Cx43 amounts on cell function. Further investigations are needed to study the interplay between the individual components of Cx43 regulation and their importance in the diseased myocardium.

## Figures and Tables

**Table 1:** Direct modification of Cx43 expression and its effects in cardiovascular cells.

Model	Intervention	Effect	Reference
Transgenic mice	Transgenic mice constitutively expressing Cx43 under control of the CMV promoter	Gap-junctional communication ↑ Postnatal viability ↓ Defects in the pulmonary outflow tract and coronary vasculature	[121]
iPSC-CMs	Adenoviral transduction or transfection of iPSC-CMs with Cx43 expression plasmid	Cx43 expression ↑ Intercellular coupling ↑ Action potential upstroke velocity ↑	[122]
Atrial fibrillation in porcine hearts	Adenoviral gene transfer of Cx43	Cx43 protein ↑ Development of persistent atrial fibrillation ↓	[123]
Atrial fibrillation in porcine hearts	Adenoviral gene transfer of Cx43	Cx43 expression ↑ Atrial conduction in sham-operated animals ↔ Atrial fibrillation ↓ Lateralization in atrial fibrillation ↓	[124]
Infarcted porcine myocardium	Injection of Cx43-expressing adenoviruses to the healed scar border	Cx43 expression ↑ Conduction velocity ↑ Ventricular tachycardia ↓	[125]
Cryoinjured mouse myocardium	Injection of Cx43-expressing lentiviruses	Cx43 expression ↑ Heterotypic cell–cell coupling ↑ Ventricular tachycardia ↓	[126]
Cryoinjured mouse myocardium	Engraftment of skeletal myoblasts from transgenic mice expressing Cx43 under control of a skeletal muscle promoter	Ventricular tachycardia ↓	[127]
Infarcted rat myocardium (permanent coronary artery ligation)	Injection of skeletal myoblasts transduced with Cx43-expressing lentivirus	Intercellular electrical coupling ↑ Arrhythmogenicity ↔	[128]
Infarcted mouse (NOD/SCID) myocardium (permanent coronary artery ligation)	Delivery of mesenchymal stem cells in combination with human skeletal myoblasts overexpressing Cx43	Left or right ventricular ejection fraction, mass of the left or right ventricle ↔	[129]
*mdx* mice	Crossing of *mdx*-mice with heterozygous Cx43-deficient mice	Normalization of Cx43 protein levels Lateralization ↓ Arrhythmia ↓	[130]
Stem cell antigen-1^+^ cells	Transfection with plasmids for expression of Cx43 fused to a mitochondrial targeting sequence	Cx43 expression ↑ Apoptosis ↓ Cell survival following oxygen–glucose deprivation ↑	[132]
Hl1-cells	Transduced to overexpress Cx43	Cx43 expression ↑ Respiration ↑	[37]
Streptozotocin-induced diabetic cardio-myopathy in mice	Overexpression of mitochondrial Cx43 using AAV2/9	Cx43 amount in mitochondria ↑ Cardiac function ↓ Susceptibility to arrhythmias ↑	[133]
Isolated mouse hearts in vitro	Ischemic preconditioning	Mitochondrial Gja1-20k ↑	[47]
	Adenoviral overexpression of Gja1-20k	ROS formation ↓ Infarct size ↓	

AAV2/9: adeno-associated virus serotype 2/9; CMV: cytomegalovirus promoter; iPSC-CM: induced pluripotent stem cell-derived cardiomyocytes; NOD/SCID: nonobese diabetic/severe combined immunodeficiency mice; *mdx* mice: mice with mutations in the dystrophin gene (a model of Duchenne muscular dystrophy); ↑: increase; ↓: decrease; ↔: unchanged.

**Table 2:** Interventions to treat cardiovascular diseases and their effects on Cx43 expression.

Model	Intervention	Effect	Reference
Porcine myocardium	I/R with ischemic preconditioning	Mitochondrial Cx43 ↑	[131]
H9c2 cells	Doxorubicin-treatment	Mitochondrial Cx43 ↑ ROS formation ↑	[134]
Isolated rat hearts in vitro	Hypothermic I/R	miR-17 ↑ Cx43 ↓	[135]
	Hypothermic I/R + inhibition of miR-17	Cx43 expression ↑ (level below control) Arrhythmia ↓	
H9c2 cells	Hypoxia/reoxygenation	Cx43 ↓	[136]
	Hypoxia/reoxygenation + lactate	Cx43 ↑ (level comparable to control)	
Rat hearts in vivo	ICM by coronary artery ligation for 4 w	Cx43 ↓	[137]
	ICM + cardiac specific overexpression of caveolin-1	Cx43 ↑ (level comparable to control)	
Isolated rat hearts in vitro	I/R	Cx43 ↓	[138]
	Injection of exosomes from C2C12 cells before I/R	Cx43 ↑ (level below control)	
Rabbit hearts in vivo	single-targeted heavy ion irradiation	Cx43 ↑ (from 2 w to 12 m after irradiation) Cx43 lateralization ↑	[139]
Rabbit hearts in vivo	Myocardial infarction by microsphere injection	Cx43 ↓	[140]
	Myocardial infarction + single targeted heavy ion irradiation after 2 w	Cx43 ↑ (level above control) Cx43 lateralization ↑ Arrhythmia ↓	
Rat hearts in vivo	Monocrotaline-induced pulmonary hypertension	Cx43 ↓	[141]
	Monocrotaline-induced pulmonary hypertension + tranilast	Cx43 ↑ (level below control) Atrial fibrillation ↓	
Rat hearts in vivo	Monocrotaline-induced pulmonary hypertension	Cx43 ↔ Lateralization ↑	[92]
	Monocrotaline-induced pulmonary hypertension + 1,8-cineole	Cx43 ↔ Lateralization ↔	
Rat pulmonary arteries	Pulmonary hypertension by chronic hypoxia in vivo	Cx43 ↑	[142]
Rat mesenteric arterioles	Spontaneously hypertensive rats	Cx43 ↑	[143]
Mouse hearts in vivo	Heart failure by transverse aortic constriction	Cx43 ↓	[144]
	Heart failure + carnosol	Cx43 ↑ (level below control) Arrhythmia ↓	
Dog hearts in vivo	Heart failure by rapid pacing	Cx43 ↓	[77]
	Heart failure + renal denervation	Cx43 ↑ (level below control)	
Mouse hearts in vivo	HFpEF by high fat diet and L-NAME HFpEF + levosimendan	Cx43 ↓	[145]
		Cx43 ↑ (level comparable to control) Mitochondrial homeostasis ↑	
Rat hearts in vivo	Cardiac remodeling by isoproterenol	Cx43 ↓	[146]
	Heart failure + carnosol	Cx43 ↑ (level comparable to control) Arrhythmia ↓	

HFpEF: heart failure with preserved ejection fraction; ICM: ischemic cardiomyopathy; I/R: ischemia/reperfusion; ROS: reactive oxygen species; ↑: increase; ↓: decrease; ↔: unchanged.

## Abbreviations

The following abbreviations are used in this manuscript:

CK1	Casein kinase 1
Cx	Connexin
DMD	Duchenne muscular dystrophy
ERAD	Endoplasmic reticulum-associated degradation
ERK	Extracellular-signal regulated kinase
H/R	hypoxia/reoxygenation
K63	Polyubiquitylation at lysine 63
LAD	Left anterior descending artery
I/R	Ischemia/reperfusion
iPSC-CMs	induced pluripotent stem cell-derived cardiomyocytes
MAPK	Mitogen-activated protein kinase
MI	Myocardial infarction
PKC	Protein kinase C
ROS	Reactive oxygen species
TAC	Transverse aortic constriction
TRPV2	Transient receptor potential vanilloid type 2
